# Syrian refugees arriving in Germany: choice of corridor and individual characteristics of forced migrants

**DOI:** 10.3389/fsoc.2023.1070065

**Published:** 2023-05-11

**Authors:** Ludger Pries, Berna Safak Zulfikar Savci

**Affiliations:** Faculty of Social Science, Ruhr University Bochum, Bochum, Germany

**Keywords:** forced migrants, migration corridors, migration trajectories, Syrian refugees, forced migration

## Abstract

In 2015 and 2016, almost two and a half million forced migrants entered the European Union. Most of them arrived in the European Union from Syria, but there were also forced migrants from Iraq, Afghanistan, and other countries. While many of these migrants used the so-called Balkan route after passing through Turkey, others arrived in Greece via Lebanon or Turkey, and some traveled via North African countries, mainly Egypt and Libya. Why did refugees use such different migration corridors? Was it a matter of economic resources, of education and knowledge, or of family ties and social networks? In this paper, we statistically analyze the migration corridors used by Syrian refugees who arrived in Germany between 2014 and 2016. Using a unique dataset of 3,125 refugees, we identify the main migration corridors used Syrian forced migrants and analyze the sociodemographic and journey-related contextual factors associated with the use of these routes. Use of different escape routes was found to correlate with person-related variables and with journey-related contextual factors. The study contributes the debate on the dynamics of forced migration and onward migration.

## 1. Introduction

During the years 2015 and 2016, the European Union (EU) registered almost two and a half million asylum applications.^1^ Most of the applicants came from Syria, but others also came from Iraq, Afghanistan, and other countries. Many traveled via the so-called Balkan route after passing through Turkey; others arrived in Greece via Lebanon or Turkey; meanwhile, other groups traveled via North African countries, mainly Egypt and Libya. Why did refugees use such different migration corridors? In order to reach the EU, e.g., from the global South, it seems to be more dangerous and more expensive than other options to travel through North African countries and cross the Mediterranean, given that between 2014 and 2022 more than 21,000 people lost their lives in the Central and Western Mediterranean—a death rate 12 times higher than in the Eastern Mediterranean (International Organization for Migration, 2022). Is it a matter of economic resources, of education and knowledge, or of family ties and social networks? In this paper, we address forced migration from Syria to the EU, especially to Germany. Although many studies exist on the dynamics of the arrival and integration of Syrian refugees in Germany and other EU member states, we know less about the specific conditions and contexts under which such refugees make use of different corridors in their migration.

Studies on the formation of migration corridors among forced migrants and their underlying decision-making processes are relatively limited. If we consider the traditional drivers of migration more generally, several sets of factors fuel the decision on where and how to migrate; these range from rational economic choices and the existence of dual labor markets to historical ties and colonial relationships. Concerning forced migration, the absence of security, violations of human rights, and the presence of (armed) criminal groups in countries of residence are the core drivers. A report by European Asylum Support Office (2016) highlights the challenge of distinguishing these political factors from economic factors and the entwinement of lack of security and economic instability as push factors. As in the case of other types of migration, the forced migration process is also not static (European Asylum Support Office, 2016); in fact, it can be a much more dynamic process than the usual forms of migration movement.

Crawley and Hagen-Zanker (2019, p. 21) analyze destination preferences and the effectiveness of migration control for 250 Syrian, Eritrean, and Nigerian migrants and explain the determinants of the migration decision as follows: “…there is strong evidence that migrant decision-making is a dynamic process, one which is influenced and shaped by a complex interaction between macro, meso, and micro level factors including historical and geographical ties between origin and destination countries and the economic, political and social resources that refugees and other migrants are able to mobilize.” Recent studies underline the non-linear character of migration, and this also holds for forced migration. Erdal et al. (2023) question the concept of “the destination” based on semi-structured interviews with 30 nurses. Against the classic assumption of a linear, clearly defined plan for migration, Crawley and Jones (2021) argue that experiences gained during the course of migration and in the places of arrival are crucial and frequently alter migrants' original ideas. Migration trajectories are more complicated and multi-layered than is assumed under traditional approaches (Crawley and Jones, 2021; Della Puppa et al., 2021; Formenti, 2023). We should analyze forced migration trajectories as segmented and fragmented or as a dynamic ongoing process (Della Puppa et al., 2021; Ahrens and King, 2023).

By considering the concept of onward migration, scholars have provided a better understanding of the complex and dynamic structure of migration trajectories. Ahrens and King (2023, p. 5) define onward migration as “…a spatial trajectory that involves extended stays in two or more destination countries. Acknowledging that any migrant can be a potential onward (or return) migrant allows for a more open-ended and processual understanding of migration. After living in one destination country, migrants may decide to move to one or more new destinations. Countries and places thus can change from being destinations to becoming points of departure.” However, Della Puppa et al. (2021) conceptualize onward migration within a narrow framework by focusing on the reactivation of intra-European migration movement among third-country nationals living in the EU under secure legal status. In their study, various forms of capital (such as economic, social, and cultural^2^) and their impact on migration trajectories are discussed; migration capital can be considered as a set of potential and actual dividends, such as knowledge of migration-related processes, EU citizenship, or permanent settlement rights, acquired through migration experiences. In this paper, we examine the characteristics of migrants instead of analyzing forms of capital, but we pay special attention to migrants' resources.

Crawley et al. (2018) differentiate between the primary drivers of forced migrants' motives to leave their country of origin, and secondary drivers that are relevant in onward migration (Crawley et al., 2018). Reasons for onward migration among forced migrants are feelings of security, inadequate employment opportunities, and the need for a sense of future (Crawley et al., 2018). Analyzing the return aspirations of Syrian refugees in Turkey, Kayaoglu et al. (2022) emphasize security, regime changes, and opportunities for a better life in Syria as elements of onward migration. Additionally, experiences of discrimination, the feeling of occasionally being exposed to discrimination, and socio-cultural distance are considered to influence return migration. A study based on qualitative and quantitative primary data from Syrians living in Turkey and Lebanon emphasizes family obligations, household income, political factors, and discrimination in the current country of residence as the main elements of desires to return (Müller-Funk and Fransen, 2020). Rottmann and Kaya (2021) explain how migration experiences in the transit country influence the migration aspirations of Istanbul's Syrians, and they underline the relevance of relations between culture, religion, and gender on the biographical projects of migrants. Topgül and Adali (2021) analyze internal onward migration among Syrian women migrants in Turkey, and show that year of arrival and choice of the first place of settlement in Turkey strongly influence onward movement within Turkey. In analysis of forced migration and onward migration, another frequently discussed element is the impact of border regimes and migration policies. Crawley and Hagen-Zanker (2019) emphasize that state efforts to reduce immigration seldom determine the preferred destinations. In their study, a mix of several elements, including access to protection, family reunification, information availability, economic climate, and social networks, are identified as drivers of destination selection. Additionally, perceptions of migration policies may matter more than their actual content, since their ramifications are often unknown or misinterpreted (Crawley and Hagen-Zanker, 2019). All of these aspects of aspirations play a role in the formation of the routes taken by forced migrants.

In this study, which is based on a unique dataset, we analyze the migration trajectories of 3,125 Syrian refugees who started their journey to Germany between 2014 and 2016.^3^ In this study, by considering migration by forced migrants not as a one-time decision, but as a process and a prolonged part of the life-course, we sought answers to the following questions. Which typical corridors of forced migration could we identify? How are variables such as time and cost of the journey and means of transportation interrelated with these corridors? How are these variables related to socio-demographic characteristics, such as number of household members, family status before migration, level of education, gender, and religious or ethnic ascriptions? By analyzing a broad person- and household-related dataset, we integrate socio-demographic facts with subjective experiences. Based on current research, we were able to derive six hypotheses that we considered relevant to test: (1) forced migrants with greater personal resources may choose a more direct route; (2) forced migrants who begin their migration journey earlier may select a more direct route; (3) the longer the route, the more expensive it may be; (4) forced migrants will choose their arrival countries according to the economic conditions of their desired destination; (5) forced migrants will choose their arrival countries according to social capital—i.e., where relatives/friends already live; and (6) forced migrants will choose their arrival countries according to the general sociocultural image of the desired destination. In the following sections of this article, we first summarize the important findings of recent studies that have focused on refugees under conditions of prolonged escape using the method of secondary analysis. We focus especially on Syrian refugees in Turkey, based on existing studies (Section 1). We then sketch out the general characteristics of refugee movement from Syria into the EU since the 2010's (Section 2). Based on these findings, we analyze a broad dataset based on surveys of Syrian refugees who started their journey to Germany between 2014 and 2016 and were surveyed in Germany between 2016 and 2017 (Section 3). The article ends with some concluding reflections on forced migration as a prolonged stage in the life-course and desiderata for further research (Section 4).

## 2. The general landscape of forced migration

As of 2021, the United Nations High Commissioner for Refugees (UNHCR) counts some 97 million people of concern, including some 21 million international refugees and 49 million internally displaced persons (IDP), 4.8 million asylum seekers, 6.2 million returned asylum seekers and IDPs, and more than 12 million other persons of concern (United Nations High Commissions for Refugees, 2021). From 2010 to 2020, the growth in the world population was ~12%, international migration increased by 27%, and forced migration (counted as persons of concern as defined by the UNHCR) grew by 123% (United Nations High Commissions for Refugees, 2010, 2021; Migration Data Portal, 2021).^4^ Beside this increasing relevance, the challenges of forced migration are concentrated in certain places and in specific social groups. In 2019, some two-thirds of all refugees came from Syria, Afghanistan, South Sudan, Myanmar, and Somalia. These refugees mainly do not travel to the EU, but are hosted in Turkey, Pakistan, Uganda, and Sudan (United Nations High Commissions for Refugees, 2020a, p. 3; for international displacement: p. 6–10; for refugees: p. 18–20.) Meanwhile, in 2015, refugees hosted in Lebanon, Jordan, or Turkey represented between 5 and 16% of the population, while in Germany—as a quite rich country—in the same year, the proportion was just one percent. Although Germany received the fifth-highest number of arriving forced migrants in 2015, in 2019 the number of first-time asylum applicants fell to 142,400, and the EU as a whole received only 612,700 first-time applicants, which represents only 2.5% of all international refugees worldwide (EUROSTAT, 2020).

In countries like Somalia, Nigeria, the Democratic Republic of the Congo, and Chad, organized violence and armed conflicts, together with weak states and insufficient public authority and security, fuel migratory processes. Since 2010/11, the so-called Arab Spring and various forms of violence (whether attributed to state authorities, to organized crime, or to political movements) have given rise to emigration from multiple Middle Eastern and North African countries to neighboring countries as well as to European countries. During the past two decades, organized violence has emerged as a key factor fueling mixed migration. This term refers to the use of physical force in a collective and organized way in order to achieve collective goals, and it can be executed in the form of state, political, criminal, or paramilitary (state-sponsored) violence, as well as including non-state-sponsored group violence committed by religious and ethnic organizations, terrorist groups, and criminal gangs engaged in kidnapping, armed robbery, reprisal attacks, rape, and murder.^5^

This context is relevant for understanding the situation of forced migrants from Syria, who had to find a way out of their country, travel through other countries, and find a secure place. Concerning the Middle East, Syria was one of the countries that hosted the greatest number of refugees and asylum seekers worldwide until 2010. After this point, the country became the state with the largest numbers of IDPs (some 6.6 million) and refugees abroad (some 6.3 million; International Displacement Monitoring Centre, 2020; United Nations High Commissions for Refugees, 2020a). Since 2014, armed organized groups and terrorist groups like *Islamic State* and *Syrian Democratic Forces* have taken control of certain areas. As a result, from 2011 onwards, in addition to the IDPs, some 5.6 million refugees moved from Syria to Turkey, Lebanon, and Jordan. Two main escape corridors, understood as clusters of individual migration routes, stabilized. One led to the north, for refugees intending to stay in Turkey or travel on toward the EU. Due to historical ties, the border between Turkey and Syria has always been easy to cross. Syrian migrants first arrived in the southern cities of Turkey, and later in its metropolitan cities. In 2019, the number of Syrian forced immigrants living in Turkey under temporary protection, including Syrian asylum seekers with pending cases, had reached 3.9 million persons of concern (United Nations High Commissions for Refugees, 2020a). As Syrians had no opportunities to flee to Israel, Palestine, or many Arabian countries (these countries did not accept them), the second refugee corridor opened to several other Arabian and North African countries, such as Lebanon, Jordan, Iraq, Egypt, and Algeria (Hudson, 2018). In 2020, with 865,531 Syrian migrants, Lebanon hosted the greatest number of refugees within this migration corridor (United Nations High Commissions for Refugees, 2020b). Almost 40% of these refugees live in Bekaa, some 27% in North Lebanon, and 24% in Beirut. The second most relevant country on this migration line is Jordan, with some 12% of the total Syrian forced migrants; this is followed by some 242,000 Syrian externally displaced persons living in Iraq and some 130,000 in Egypt.

When the conflict in Syria began in 2011, migration patterns indicated that Turkey was a transit country. Legislation and institutional infrastructure were designed neither for immigrants nor for refugees or asylum seekers. Two legal regulations were of significance for forced migrants. The first was the 1951 Geneva Convention and the 1967 Additional Protocol on the status of refugees; however, Turkey accepted the corresponding stipulations only with respect to European citizens. The second was the 1994 Regulation on Asylum, the first legal document regulating asylum issues, which was prepared in light of forced migration from Iraq to Turkey during the early 1990's (Içduygu, 2015).^6^ When the first group of 252 Syrian forced migrants arrived in Turkey in April 2011, Turkey had no inclusive legal regulation for immigrants and refugees; however, during 2011, Turkey began to adopt an open-door policy (Içduygu, 2015; Erdogan, 2020).

In October 2011, at UN meetings in Geneva, the Turkish government announced, as a first step, a policy of *temporary protection* for Syrians. A second announcement declared that incoming forced migrants would be treated according to the *principle of non-refoulement*, which means not sending anyone back without their request. The third component announced was the promise to provide for the basic human needs of those who had escaped from the violent environment in Syria (Kirisci, 2013; Içduygu, 2015; Kaya, 2020). At the end of 2019, the United Nations High Commissions for Refugees (2020a, p. 75) documented 3,579,531 refugees and people in refugee-like situations, as well as 328,257 asylum seekers with pending cases. In 2020 alone, 31,334 migrants applied for international protection in Turkey, consisting of 72% Afghan citizens, 19% Iraqi citizens, 5% Iranian citizens, and 4% citizens of other countries (Directorate General of Migration Management, Ministry of Interior Turkey, 2021a). The number of irregular migrants, as recorded by the Directorate General of Migration Management in Turkey (DGMM), reached its highest level in 2019 (454,662); this figure also sheds light on the scale of forced migration into the country (Directorate General of Migration Management, Ministry of Interior Turkey, 2021b). Among such migrants, the majority were from Afghanistan (201,437 persons), followed by Pakistan, Syria, Palestine, and Iraq.

In sum, Turkey has played a crucial role in migration paths throughout history. Due to economic growth, it remained a transit country, although it also started to become a destination country for immigration. However, the war in Syria caused Turkey to become the host country with the most internationally displaced persons in the world (United Nations High Commissions for Refugees, 2020a). As a special feature, only 1.8% (67,000 persons) of the Syrian population in Turkey live in camps across five provinces (Erdogan, 2019). The vast majority of the Syrian population live in urban areas concentrated in the border provinces with Syria and in provinces with Turkish citizens of Arabian ancestry. Almost half of all Syrian forced migrants (a total of 1,790,669 people) live in Gaziantep, Hatay, Sanliurfa, Adana, and Mersin. Additionally, developed economic areas and regions with very large labor markets have also attracted forced migrants, especially Istanbul, which hosts 525,529 Syrian migrants (Directorate General of Migration Management, Ministry of Interior Turkey, 2021c).

Concerning the sociodemographic profiles of Syrian migrants living in Turkey, there is an inadequate registration system.^7^ According to the Turkish Demography and Health Survey (Hacettepe University Institute of Population Studies, 2019), the sex ratio of Syrians in Turkey (male:female) is 108:100. Syrians in Turkey are younger than the average age of the population in Turkey (Adali and Türkyilmaz, 2020). More than one million of the Syrians living in Turkey are under 18, and over two million are of active working age, between 15 and 64 (Erdogan, 2019). Households of Syrian forced migrants in Turkey are male-headed and consist of an average of six members (Hacettepe University Institute of Population Studies, 2019). Syrian migrants on average have lower levels of education than the Turkish population (Erdogan, 2019). During the initial years of forced migrants' arrival in Turkey, more than half of Syrians registered in camps reported their education level as a maximum of primary school; outside camps, this percentage increased to some six out of 10. The proportion who reported having a high school or higher degree was 21% within camps and 19% outside camps (Disaster and Emergency Management Presidency Ministry of Interior Turkey, 2013). At the end of the 2010's, an estimated one-third of Syrian refugees were illiterate (Erdogan, 2019). According to Erdogan (2019), the low educational levels reflect the extraction of refugees primarily from rural and traditional regions of northern Syria. Female migrants were found to have lower educational levels than male migrants (Hacettepe University Institute of Population Studies, 2019). It is thought that the 100's of 1,000's of Syrian refugees who have left Turkey—either toward the EU or back to Syria—on average have higher levels of education (Erdogan, 2019). A recent study reveals that most refugees report having had a regular job in Syria before leaving; only 17% were unemployed (Turkish Red Crescent and World Food Program, 2019).

In sum, what was thought of as a temporary escape from Syria to Turkey has often become a prolonged stay and has often led migrants to reconsider their life courses and biographical futures. This perspective also takes into account the “unanticipated consequences of purposive social action” (Merton, 1936), e.g., leaving one's home for a projected short period and then having to arrange a new life in Turkey or being invited by relatives to further migrate to another country, e.g., in the EU. Criteria and priorities for action and decision-making shift over time and have to be negotiated within broader social networks of families and friendships, as well as in the context of national and local migration regimes. As no panel studies or retrospective life trajectory surveys have yet been conducted in Turkey, it would be interesting to know whether Syrian forced migrants who arrived in the EU share the same or similar characteristics with those living in Turkey. In addition, why did they travel there and through which migration routes?

## 3. Characteristics of recently arrived Syrian refugees in Germany

The scientific literature on Syrian forced migration to Germany concentrates on the dynamics since the so-called Arab Spring in 2010. However, migration from Syria to Germany was already occurring before this point, mainly among more highly qualified Syrian elites. During the 1980's, the Syrian immigrant population living in Germany consisted mainly of students (Ragab et al., 2017; Worbs et al., 2020). From a forced migration perspective, a considerable influx from Syria to Germany occurred because of the riots in Hama in the early 1980's (Ragab et al., 2017). A second wave of forced migration then occurred in the context of the civil war in Syria after 2010. By 2010, some 30,000 Syrian immigrants already lived in Germany (Worbs et al., 2020). Over a single decade, and mainly due to organized violence and forced migration, the number of Syrians living in Germany increased to more than 780,000 in 2020 (Bundesamt für Migration und Flüchtlinge, Federal Office for Migration and Refugees, 2020).

Among first-time asylum applicants, until 2012, Kurdish ethnicity was dominant among Syrians living in Germany. After 2015 and 2016, this shifted toward persons indicating Arabic ethnicity (almost two-thirds). In terms of religious beliefs, the majority declare themselves to be Muslims, with minorities describing themselves as Catholics or Yazidis (Worbs et al., 2020). In the first wave of the IAB-BAMF-SOEP Refugee Survey, reasons given for leaving the country of origin were concentrated on the context of organized violence (“fear of violent conflict/war,” “persecution,” “fear of forced conscription”). In response to the question of why the interviewee chose Germany, almost three-quarters mentioned “respect for human rights.” Some 43% referred to the quality of the German education system, and 42% declared that they felt welcome in Germany. Smaller proportions (from one-quarter to some 12%) of respondents reported reasons relating to the welfare system or economic situation, or already having relatives, friends, or people from the same country of origin in Germany (Brücker et al., 2016). Those refugees who came to Germany directly from their country of origin reported a mean cost of 7,137 euros for transportation, housing, and border crossing.^8^ Those who spent a longer period in a transit country reported total costs, on average, of 5,231 euros. In the case of refugees from the West Balkans, Soviet Union, Syria, Iran, Iraq, Lebanon, and Palestine, almost 90% of refugees reported having arrived in Germany within only 2 years of having left their country of origin. Interestingly, the total cost, as well as the average duration of the migration trajectory, fell substantially from 2013 to 2014 and from 2014 to 2015 (Brücker et al., 2016, p. 5f).

Educational levels varied substantially according to country of origin. In general, more than 70% reported having attended middle or secondary school, and more than half of all refugees interviewed reported having graduated from these schools. Some 58% of respondents indicated that they had spent at least 10 years in formal schooling; for the overall population living in Germany, this rate is 88% (Brücker et al., 2016, 2018). Language, writing, and reading skills varied substantially between refugees by country of origin, with forced migrants from Syria ranking above average (Brücker et al., 2018, p. 34). Considering the religious affiliation of all refugees who participated in the IAB-BAMF-SOEP Refugee Survey (second wave v.34), Syrian refugees had the highest share of affiliation to Islam, whereas, e.g., the majority of refugees from Iran—also a predominantly Islamic country—described themselves as Christian (Siegert, 2020). Almost three-quarters of the refugees interviewed were male (of these, almost half had no partner in 2017); only one-quarter were female (of these, almost three-quarters had a partner). Notably, rates of mental health and depressive symptoms and risk of post-traumatic stress disorder are substantially higher in refugees than the general population in Germany (Brücker et al., 2019).

Examining the Syrian population living in Germany in general, according to data from the Federal Office of Statistics, in 2017 some two-thirds of this population were men. This is 12% higher than the average share of men among all foreigners living in Germany and reflects the high proportion of refugees in the overall Syrian population (Worbs et al., 2016). As in Turkey, the Syrian refugee population in Germany is quite young, with an average age of 24.2 years in 2017 and a share of persons aged up to 15 of almost one-third (Worbs et al., 2020); by comparison, this share was 40% in Turkey in 2020 (see Directorate General of Migration Management, Ministry of Interior Turkey, 2021c). In fact, with an average age of 38 years, Syrians in Germany in general are younger than the overall population of foreigners in Germany. Because of the age-selective nature of Syrian migration to Germany, a large proportion of Syrians continue in the education system (Ragab et al., 2017).

Studies show that the population of Syrian forced migrants in Germany is male-dominated (some 70% being male in 2015 and 61% in 2017; Juran and Broer, 2017; Worbs et al., 2020). Considering the marital status of Syrians in Germany at the end of 2017, 58% reported being single (63% of Syrian men and 51% of Syrian women). At the end of 2017, only 31% of Syrians were married, whereas this proportion was 43% among all foreign residents (Worbs et al., 2020). Until 2015, Syrians mostly lived in North Rhine-Westphalia, Lower Saxony, Bavaria, and Baden-Wuerttemberg (Ragab et al., 2017). The distribution quota system among asylum seekers and refugees subsequently led to a more balanced geographical distribution of Syrians (Worbs et al., 2020). Although the education level of Syrian refugees is lower than that of the overall residents in Germany, it is higher than that of other refugee groups (Worbs et al., 2020, p. 221). The share of Syrian first-time applicants with no formal education was 3%, compared to some 8% among all first-time asylum applicants (Ragab et al., 2017, p. 21).^9^ The pattern of gender differences in education level among Syrian forced migrants in Germany is similar to the pattern seen in Turkey, with women having lower levels of education overall (Worbs et al., 2020). Employment opportunities for refugees are an integral component of integration efforts, but finding employment is more challenging than for native citizens in OECD countries (Organization for Economic Cooperation and Development, 2017). In addition, their living standards are directly related to their employment circumstances. Worbs et al. (2020) calculated that in 2017 the average income among Syrians was < 60% of the median income in Germany, meaning that 80% were living at risk of poverty. In the same year, some 9% of all job-seekers in Germany were forced migrants (Organization for Economic Cooperation and Development, 2017).

We can conclude that, in the case of both countries, forced migration is often not simply a brief event in the life course followed by a unidirectional integration process in a new country. Forced migration needs to be studied as prolonged social practice and a form of everyday life, in which people must negotiate and reconstruct their aims, identities, and framings of their biographical projects (Pries, 2018, chapters 6 and 7). Given these commonalities and differences, it is to be expected that forced migrants from Syria seek out different and new geographic routes and corridors by which to migrate. They might plan to transit through Turkey and become stuck in that country; or, conversely, they might plan with the aim of staying in Turkey and then decide to move on. During 2015 and 2016, the refugee movement from Syria triggered the creation of new migration routes (Yüceşahin and Sirkeci, 2017; Hudson, 2018), and this was not mainly prompted by Merkel's famous statement “We will manage it” (Pries, 2019). One important question is by which routes the Syrian refugees came to Germany and why they did so. Are there particular features of different migration routes and of the individuals using them? In addition to the route across the Aegean Sea to Greece, there was also the Balkan route and the route from North Africa to Italy. Who takes which route and why? As demonstrated elsewhere, the routes of forced migrants toward the EU vary in time and space according to many factors. To some extent, these factors are related to the characteristics, motivations, and experiences of the forced migrants themselves; on the other hand, they are also dependent on macro-level drivers, such as the political conditions, migration policies and regimes, and the obstacles and risks found on the routes. In particular, border restrictions normally do not stop migration movements, but redirect them toward other corridors (for refugee immigration to the EU, see Pries, 2018, chapter 4). Which factors influence refugees' choices of individual escape routes, thereby forming the basis of clustered corridors?

## 4. Migration corridors used by Syrian refugees to Germany

The following analysis of the migration trajectories of Syrian forced migrants to Germany is based on a unique dataset. The Institute for Employment Research (IAB), the Research Center of the Federal Office for Migration and Refugees (BAMF-FZ), and the German Socio-Economic Panel (SOEP) at the German Institute for Economic Research (DIW Berlin) jointly initiated a representative and detailed panel survey. Between June and October 2016, an initial wave of interviews with 2,349 individuals from 1,766 households were conducted; later in 2016, a further 2,467 individuals were interviewed. In 2017, a total of 4,525 individuals were interviewed; due to interview problems and quality checks, this number was reduced to 4,463 for this second wave of the panel carried out in 2017.^10^ Only adults older than 18 years of age were interviewed, but data were also registered for minors in the corresponding households. The study provides information on the drivers of forced migration decisions, routes of travel, sociodemographic characteristics, life history in terms of education and employment, family composition, accommodation conditions, asylum procedure status, and subjective values and orientations.^11^ The IAB-BAMF-SOEP Refugee Survey has provided the most comprehensive and representative dataset in the area of forced migration.

The survey includes retrospective questions relating to earlier stages of the respondent's forced migration journey to Germany; nevertheless, it provides data only on the beginning and end dates of all migration stays, in cases where the interviewee spent a minimum of 3 months in a particular location. This rather extended period impedes identification of all the places that migrants have passed through on their journeys to Germany. However, based on the information about these trajectories and the corresponding sequences of countries that the refugees had passed through, as a first step, 10 different migration corridors can be identified (Table 1).^12^ As expected, most (59%) of the people from Syria surveyed, all of whom had started their journey between 2014 and 2016 and subsequently arrived in Germany, reported a migration journey of < 3 months' duration and did not report a residential stay of < 3 months during their flight. Another considerable proportion (18%) reported having lived in Turkey for at least 3 months before arriving in Germany. More than 200 of the people interviewed (7%) indicated that they had taken a route that included a stay of at least 3 months in Lebanon or Iraq before coming to Germany. In addition to these three types of case, covering the vast majority of interviewees (some 84%), there were quite a considerable number of people who reported more than one residential stay of a minimum of 3 months before arriving in Germany. Similarly large numbers were counted for the Syria–Iraq or Lebanon–Germany route and for a journey via the Middle East and North African (MENA) region, now with a minimum of two residential stays in each case. The remainder of the interviewees (almost a tenth) reported having taken rather complex routes from Syria to Germany, including a minimum of two residential stays in more than one country.

**Table 1:** Main routes taken by Syrian forced migrants.

**Main route**	**Total number**	**Cum. Percantage**
Syria—Germany	1,828	58.5
Syria—Turkey—Germany	552	17.7
Syria—Iraq or Lebanon—Germany	217	6.9
Syria—MENA countries—Germany	191	6.1
Syria—Turkey—EU and Balkan countries—Germany	46	1.5
Syria—EU countries—Germany	47	1.5
Syria—Balkan countries—Germany	40	1.3
Syria—MENA—EU and Turkey—Germany	38	1.2
Double or triple complex routes	86	2.8
Complex circular routes	80	2.6
Overall	3,125	100.0

Source: own analysis based on the IAB-BAMF-SOEP Refugee Survey v.34.

**Figure 1:**
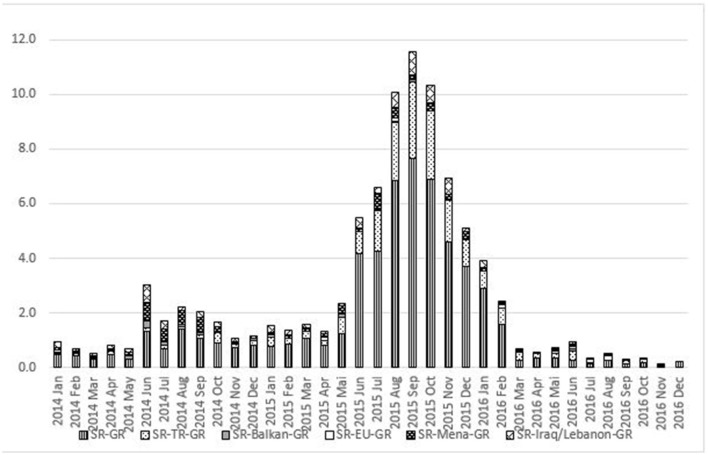
Corridor selection by month of leaving Syria. Source: own analysis based on the IAB-BAMF-SOEP Refugee Survey v.34.

How might we explain these very different trajectories followed by forced migrants? Does use of the different corridors vary according to the time at which an individual's migration from Syria started? This could be the case when, e.g., some escape routes have closed or became more challenging. For instance, during 2015 and beginning of 2016, the land borders between Turkey and Greece and between Turkey and Bulgaria and the sea border between Turkey and Greece happened to be either closed and strongly controlled or quite easy to cross. The same holds during other periods for the MENA–Italy route, etc. Therefore, we conducted an analysis based on the month when each refugee started their emigration from Syria, differentiating between the six most common corridors. Based on the second wave of the IAB-BAMF-SOEP Refugee Survey (v.34), Figure 1 indicates the share of respondents who left Syria by a certain route each month as a percentage of the total number of refugees from Syria in the overall period (January 2014 to December 2016). To reduce the complexity of examining many different routes, we analyzed only six main corridors (Syria–Germany, Syria–Turkey–Germany, Syria–Balkans–Germany, Syria–EU countries–Germany, Syria–MENA countries–Germany, and Syria–Iraq/Lebanon–Germany). As can be seen, the two routes Syria–Balkans–Germany and Syria–EU countries–Germany (in terms of a stay of at least 3 months mid-trajectory) were not so important in relative terms.

The sequence of columns shown in Figure 1 reveals a certain seasonal dynamic for 2014 and 2015, with the summer months (here June to October) being the peak season for flight. Similar seasonal waves can also be observed for all other corridors of forced migration into the EU over many years (showing more flight occurring during the warmer summer months).^13^ However, the substantial increase in June 2014 (as shown in Figure 1) must be explained by other events. On 3 June 2014, a presidential election was held in Syria, and 1 day later, the Syrian government declared President Assad to be the winner; meanwhile, western countries believed that the election was manipulated and the result meaningless. On 29 June 2014, the so-called Islamic State proclaimed its own Caliphate on the territory of Iraq and Syria and began to distribute its own currency. From then onward, repression by the Syrian Assad government, totalitarian control of some regions by the so-called Islamic State, and complex armed conflicts between different groups involved in organized violence intensified all over the northern parts of Syria.^14^ In sum, the forced migration that occurred in 2014 and 2015 has to be explained in the context of the turbulent situation with respect to organized violence. Looking only at the period from June to December 2015, almost a third of all surveyed Syrian refugees, who left the country between January 2014 and December 2016, made their way directly to Germany without any residential stop (lowest part of the stacked columns). During the same period, more than a tenth of all interviewees reported having first fled to Turkey and stayed there for a minimum of 3 months before arriving in Germany (second part of the stacked columns). The other four corridors were of less quantitative weight, with the route via Lebanon or Iraq being the third most important during the months of August to November 2016.

As outlined in Figure 1, according to the representative data, a mass emigration from Syria began in June 2015. Of interviewees who began their journey that month, 130 (or 4% of all interviewees) took a direct migration route from Syria to Germany without any intermediate stay longer than 3 months. Including the other five routes shown in Figure 1, some 6% of all migrants interviewed started their journey during this month. Considering all routes, the period between June 2015 and February 2016 (with the addition of June 2014) was when the highest rates of monthly departures from Syria occurred. The data also suggest that there was no direct impact of the highly debated statement by Germany's Chancellor Merkel, made in August 2015, that “We will manage it,” given that the highest rate of growth in emigration across all routes occurred from June to August 2015. Merkel's press conference in which she made the famous statement took place on the last day of August; therefore, it could have taken effect only from September onward (for a deeper discussion, see Pries, 2019). As can be seen in Figure 1, the rate of flight from Syria to Germany accelerated from June 2015 until February 2016, and all routes expanded in volume of use during this period.

Can we identify specific variables that explain the use of such different corridors of refuge as (on the one hand) migrating directly from Syria to Germany within less than a quarter of a year and (on the other) migrating via long distances, sometimes traveling back and forth (as in the case of fleeing from Syria initially for a long stay in Iraq or Lebanon), and then continuing onward to Germany? Why did some forced migrants take a route via North Africa? Could we explain this with reference to the time taken or the costs associated with the different corridors? For deeper analysis, we grouped the corridors into five categories according to their numerical relevance: Syria–Germany, Syria–Turkey–Germany, Syria–MENA countries–Germany, Syria–Iraq/Lebanon–Germany, and Other Routes. Table 2 presents the average time (in days) spent in transit after leaving the last country of stay, as well as the money spent in traveling via these different corridors. As expected, the routes via Iraq and Lebanon and through other Middle Eastern or North African countries took significantly longer than the direct route from Syria to Germany or the route following a long stay in Turkey. Even the category of “other routes,” which often included circular or back-and-forth migration, on average lasted fewer days than the SR-MENA-GR and SR-Leb./Iraq-GR routes. Although the difference between routes was statistically significant, the correlation between corridor taken and mean time taken was relatively weak (η^2^ = 0.013).

**Table 2:** Average time (in days) and money (in Euros) spent according to type of corridor taken.

**Corridor**	Average time^***^	**Transportation costs**	Accommodation costs^*^	**Escape/smuggling fees**	**Total costs**
	***n*** = **1,751**	***n*** = **582**	***n*** = **255**	***n*** = **461**	***n*** = **759**
SR-GR	35.6	3,921	1,312	4,367	**6,214**
SR-TR-GR	35.2	3,758	1,701	4,141	**5,802**
SR-MENA-GR	66.2	3,185	1,620	2,963	**4,643**
SR-Leb./Iraq-GR	67.8	4,017	3,110	4,602	**6,668**
Other	53.9	4,072	2,684	4,843	**6,865**
**Overall**	**42.5**	**3,864**	**1,646**	**4,324**	**6,142**

Source: own analysis based on the IAB-BAMF-SOEP Refugee Survey v.34.

* Statistically significant variance at 90% level.

*** Statistically significant variance at 99% level.

When considering the costs of different corridors, we only found an association of corridor taken with the cost of accommodation, and this was only at a low 90% confidence level. Interestingly, the *types of costs (including time)* varied significantly with the different corridors. This indicates that there seems to be a certain trade-off in the characteristics of the different kinds of route: where costs for smuggling and “professional services” were high, costs for transportation and accommodation appear to have been low, and vice versa. If we assume transparent and intensive communication (Pries, 2018) about these issues relating to the time taken and costs of migration, it is surprising that some groups of forced migrants choose an expensive and time-consuming corridor while others opt for a shorter and cheaper one. Chi-squared tests of independence indicated no evidence for an association between route and occupational status (χ^2^ = 7.786, *p* = 0.802), or between route and social status in the migrant's country of origin (χ^2^ = 16.735, *p* = 0.403). In contrast, a chi-squared test of independence examining the association between method of financing of the forced migration and type of route taken was statistically significant (χ^2^ = 147.135, *p* = 0.001). Which factors can help us to understand and explain these different action strategies? Certain survey questions related to the context in which the forced migration trajectory was initiated, some basic factors relating to experiences during flight, and the reasons for choosing Germany as a destination country could provide a better understanding.

**Figure 2:**
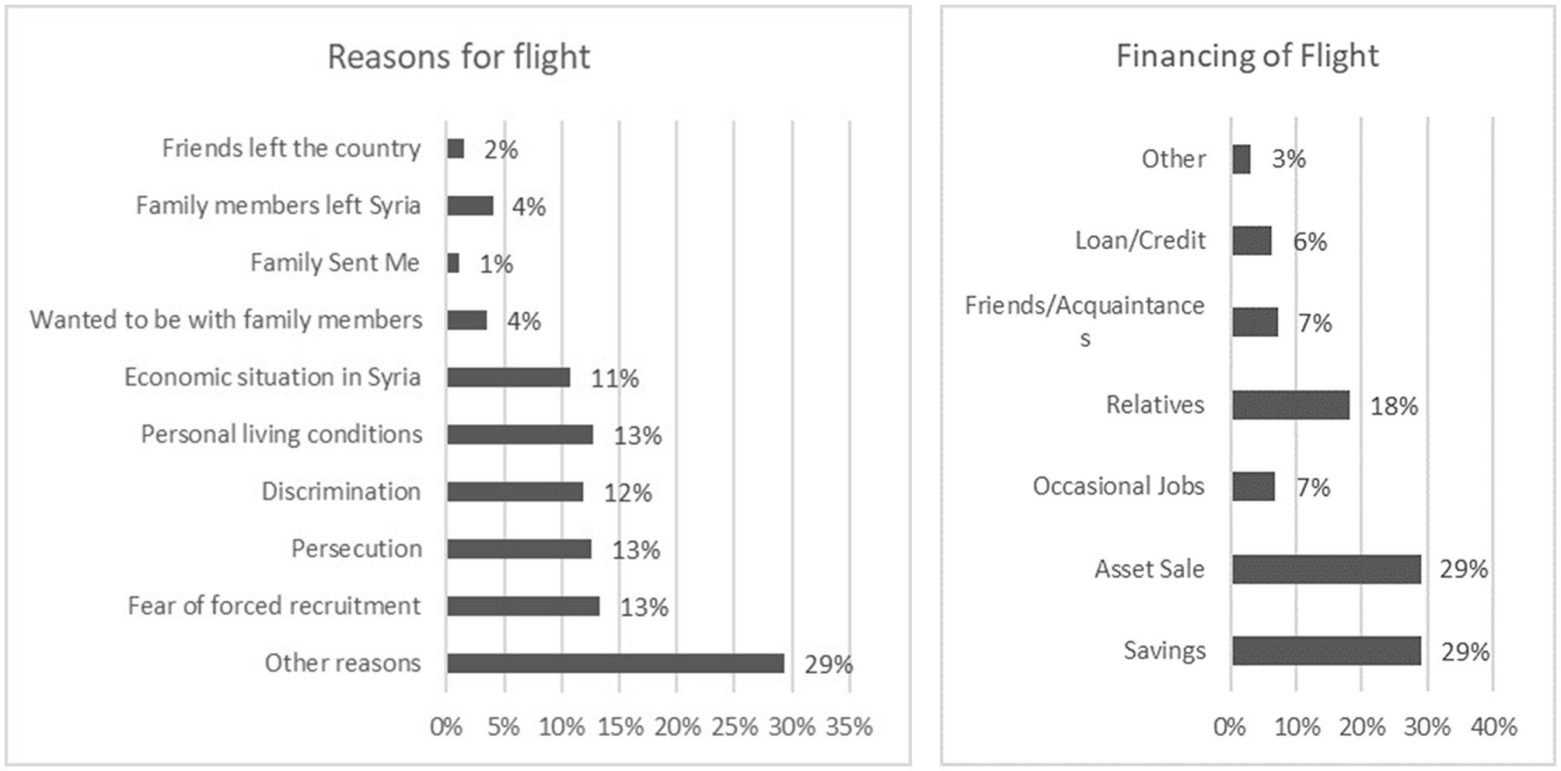
Context in which forced migration was initiated. Source: own analysis based on the IAB-BAMF-SOEP Refugee Survey v.34.

Figure 2 presents the frequencies of answers concerning the reasons for and financing of forced migration. Five main reasons stand out, and these could be classified into two main categories: violence-based reasons and economic reasons. Thirteen percent of responses indicated “fear of forced recruitment” in Syria, followed by persecution (13%) and discrimination (12%). In terms of economic reasons, the economic situation of the country and the respondent's personal living conditions were most frequently mentioned (11 and 13%, respectively).^15^ Concerning the resources used for financing the flight, the three most frequently mentioned sources were savings (29%), assets sold (29%), and money from relatives (18%).^16^

**Figure 3:**
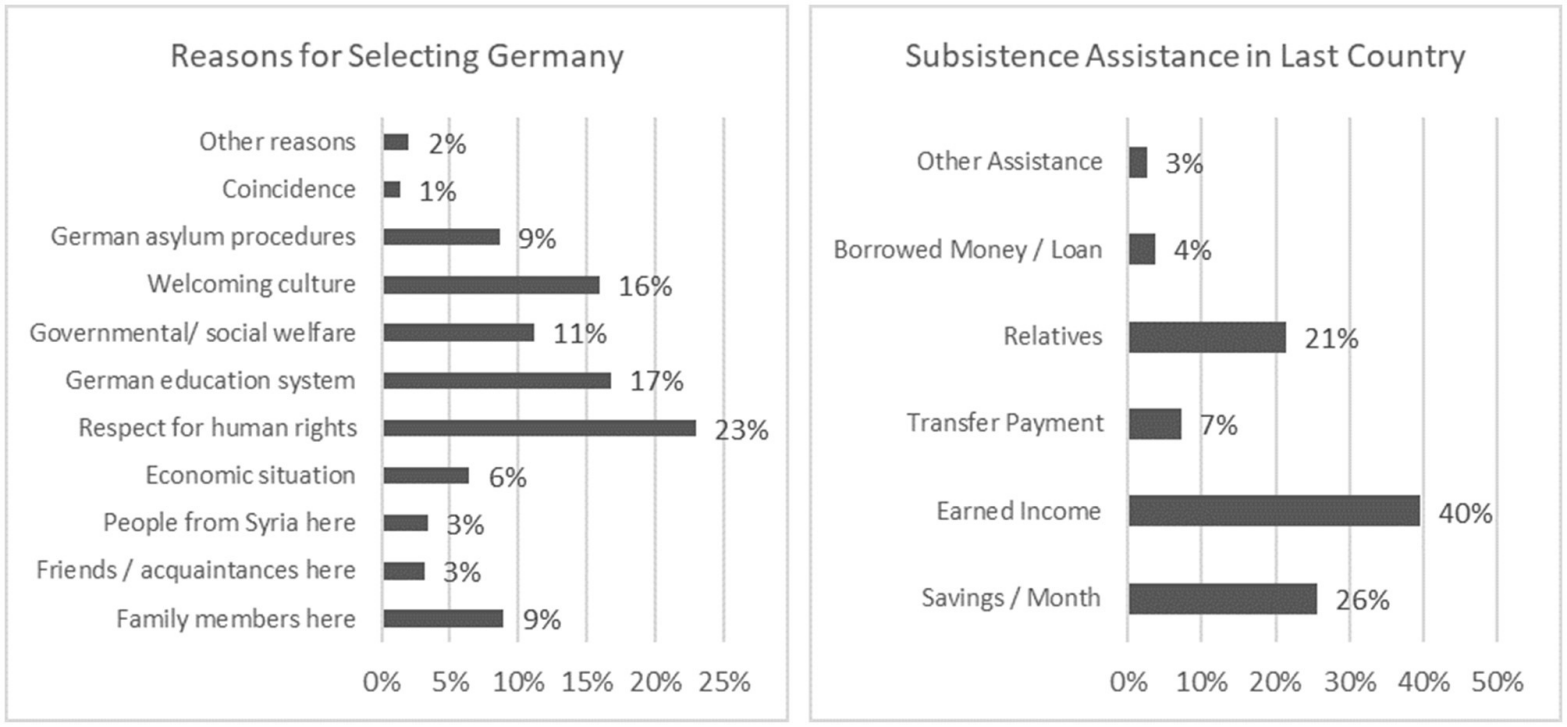
Context and aspects of migrants' flight. Source: own analysis based on the IAB-BAMF-SOEP Refugee Survey v.34.

During flight, migrants can be very vulnerable and may be compelled to experience many adverse circumstances. Among all responses to a question about the most important negative experiences, 20% of responses indicated fraud and exploitation, and the second most frequent response was incarceration (14%). Another question related to the means by which respondents had made a living in their last country of residence during flight (Figure 3). Most responses (40%) indicated that the respondent had earned an income in the country of transit; the second most common answer (26%) was living on savings; and the third (21%) was support from relatives.^17^ In the survey, migrants also were asked about their future plans at the time when they were staying for 3 months or longer in another country before arriving in Germany. The most frequent answer (67%) was that they planned to move from there to another country as soon as possible, while 16% of interviewees planned to stay where they were for longer, and 17% reported that they planned to return to their country as soon as possible. This illustrates the iterative and sequential nature of forced migration: one-third of the interviewees changed their plans (to stay longer in a transit country or return to their country of origin) in moving to Germany.

Why did migrants choose Germany as their destination country? The most frequently mentioned reason (23% of all responses) was “respect for human rights,” followed by the education system in Germany (17%) and the perception of a welcoming culture (16%). A reason less often given (11%) was the governmental and social welfare system.^18^ Other reasons, such as the German procedural system for asylum, the general economic conditions of the country, or having family members already living in Germany, were of minor relevance in the answers. All these data illustrate the nature of flight as an ongoing, protracted period in the life course. Forced migrants must adapt to new conditions in transit countries. They might find (informal) jobs to sustain their income or be dependent on their existing savings or on relatives. They might experience fraud, discrimination, and exploitation. All these factors, in addition to the migration plans of family members and relatives, must be negotiated and balanced. For these reasons, forced migration represents an extended period of social practice and everyday life under highly volatile and challenging conditions.

How can we relate this social practice of forced migration to the different corridors used by Syrian refugees? Additionally, (how) are these corridors related to individuals' specific sociodemographic characteristics? Is there any association between the formation of the corridors and the time at which an individual started their journey? Based on the IAB-BAMF-SOEP Refugee Survey, Table 3 offers insights into the association between migrant routes and the sociodemographic characteristics of refugees. Five main aggregated migration routes were considered, according to their frequency: Syria–Germany, Syria–Turkey–Germany route, Syria–MENA countries–Germany, Syria–Lebanon/Iraq–Germany, and other routes, including complex routes. In terms of sociodemographic characteristics, number of years spent in education, marital status, number of children, and mother tongue were taken into account. Concerning the time period at which each journey started, we focused on political turning points, such as the election in Syria and the signing of the EU–Turkey deal. We considered four time periods from January 2014 to the end of December 2016.

**Table 3:** Migration corridors and sociodemographic variables.

**Migration corridor factors**	**SR–GR**	**SR–TR–GR**	**SR–MENA– GR**	**SR–Lebanon/Iraq–GR**	**Other routes**
**Number of years in education** ^***^					
1–5 years	47.9%	15.5%	12.7%	11.3%	12.7%
*Adjusted residuals*	*−1.2*	*−0.4*	*1.4*	*1.0*	*0.3*
6–10 years	50.8%	20.6%	8.3%	10.0%	10.3%
*Adjusted residuals*	*−2.6*	*3.0*	*0.1*	*2.2*	*−1.4*
11–15 years	59.3%	14.5%	7.2%	6.7%	12.3%
*Adjusted residuals*	*3.9*	*−3.0*	*−1.6*	*−2.3*	*0.8*
15+ years	33.3%	20.5%	20.5%	5.1%	20.5%
*Adjusted residuals*	*−2.7*	*0.6*	*2.8*	*−0.7*	*1.7*
**Marital status** ^**^					
Single	53.1%	20.9%	5.4%	8.2%	12.3%
*Adjusted residuals*	*−1.2*	*2.5*	*−2.3*	*0.1*	*0.8*
Married	56.0%	15.6%	9.0%	8.0%	11.4%
*Adjusted residuals*	*0.8*	*−2.8*	*2.7*	*−0.2*	*−0.1*
Divorced and widowed	60.4%	22.6%	3.8%	9.4%	3.8%
*Adjusted residuals*	*0.7*	*1.1*	*−1.1*	*0.4*	*−1.8*
**Children (not significant)**					
Yes	55.4%	16.2%	8.7%	8.3%	11.4%
*Adjusted residuals*	*0.1*	*−1.6*	*1.6*	*0.5*	*−0.1*
No	55.2%	19.1%	6.6%	7.6%	11.5%
*Adjusted residuals*	*−0.1*	*1.6*	*−1.6*	*−0.5*	*0.1*
**Mother tongue** ^***^					
Arabic	57.2%	14.6%	10.2%	8.1%	9.9%
*Adjusted residuals*	*2.8*	*−5.7*	*6.4*	*−0.1*	*−2.9*
Kurdish	49.5%	26.6%	0.7%	8.3%	14.9%
*Adjusted residuals*	*−2.8*	*5.7*	*−6.4*	*0.1*	*2.9*
**Timing of the start of migration journey** ^***^					
January 2014 to May 2014	45.5%	8.2%	8.2%	13.4%	24.6%
*Adjusted residuals*	*−3.1*	*−2.9*	*1.0*	*3.0*	*5.3*
June 2014 to May 2015	50%	11.5%	13.7%	10.5%	14.3%
*Adjusted residuals*	*−5.3*	*−5.0*	*9.7*	*4.3*	*3.5*
June 2015 to February 2016	63.7%	20%	3.3%	5.5%	7.5%
*Adjusted residuals*	*8.5*	*4.9*	*−9.5*	*−4.5*	*−8.5*
March 2016 to December 2016	40.9%	22.8%	7.0%	4.1%	25.1%
*Adjusted residuals*	*−4.8*	*1.8*	*0.5*	*−1.5*	*6.2*

Source: own analysis based on the IAB-BAMF-SOEP Refugee Survey v.34.

** Statistically significant variance at 95% level.

*** Statistically significant variance at 99% level.

In our analysis of the association between education level and migration corridor used, education was operationalized as the grouped number of years spent in formal education. The vast majority of interviewees (93%) indicated that they had received between 6 and 15 years of schooling. A chi-squared test (*n* = 1,662) indicated a significant association between number of years spent in education and migration corridor used. Analysis of the adjusted residuals^19^ as a measure of each cell's contribution to the chi-squared value revealed that, e.g., migrants with 11–15 years of schooling used the Syria–Germany migration corridor more often than other routes.^20^ Users of the SR–MENA–GR corridor were polarized, with a tendency to have either few or many years of education. Additionally, the marital status variable (*n* = 1,807) had a significant association with choice of corridor. Single individuals used the SR–TR–GR route more often than expected and the SR–MENA–GR route less often than expected. In the case of interviewees who reported being married, these frequencies were the other way round. Interestingly, we did not have enough evidence to determine any association between having children and choice of migration corridor.

Taking an individual's mother tongue as an indicator of ethnic group, in the sample (*n* = 1,768) there was a significant association between ethnic group and choice of corridor. Those indicating Kurdish as their mother tongue indicated having chosen the SR–TR–GR route more frequently than expected. Concerning the time period during which refugees started their journeys (*n* = 3,125), the majority (67%) did so between June 2015 and February 2016. A chi-squared test of the association between timing and corridor used revealed a significant association between these variables. The adjusted residuals indicated that refugees who started their journey between January 2014 and May 2014 more often selected routes falling into the category of “other routes,” which also covered cases of return or circular migration. Interviewees who left during the period between the election in Syria and May 2015 selected the SR–MENA–GR corridor more frequently than expected. Finally, during the period between the vast influx of migrants (starting from June 2015) and the month before the signing of the EU–Turkey deal (March 2016), the SR–GR and SR–TR–GR corridors were selected more frequently than expected.

## 5. Conclusion: forced migration as protracted stage in the life-course

Much of the current attention in studies of refugees focuses on the dynamics of arrival and integration of refugees. Here, we have concentrated on shedding light on forced migration, not as a one-time decision, but as a process and a prolonged component of the life course and social practice of forced migrants. We have highlighted the often-unintended consequences of “temporary” escape from Syria to Turkey. In addition, we have compared the sociodemographic characteristics of those forced migrants with those of Syrian refugees who started their journey to Germany between 2014 and 2016. Based on the large dataset from the IAB-BAMF-SOEP Refugee Survey, we identified different types of corridors used by those fleeing from Syria. Based on clustering of individual migration routes into corridors of refuge, we analyzed the factors that correlate with use of these different corridors.

Concerning our guiding hypotheses, we did not have enough evidence to assert that (1) forced migrants with greater personal resources may choose a more direct route. In particular, chi-squared tests of independence could not provide us with enough information to support this hypothesis. The results of chi-squared tests of the associations between the formation of these corridors and migrants' occupational status, and between choice of corridor and social status, indicated the independence of these factors. There was a statistically significant association only in the case of one proxy variable for resources (the method of financing the escape). Concerning our hypothesis (2) that forced migrants who began their migration journey earlier may have selected a more direct route, our analyses showed that “early” forced migrants preferred to go to neighboring countries with cultural proximity. The analysis indicated that, at the earlier stages, the routes SR–Lebanon/Iraq–GR, SR–MENA–GR, and those falling under “other routes” (which also covered circular migration) were chosen more frequently than expected. Refugees might have hoped to be able to return to Syria soon. We also could not verify our hypothesis (3) that the longer a route, the more expensive it may be. The presence of the migration industry may be a determinant of the cost of travel regardless of the distance between Syria and the target destination. Hypotheses (4) to (6) related to the reasons to select Germany as a destination country. Basic descriptive analyses indicated that refugees chose their arrival countries according to their general sociocultural image. Among the group of Syrian refugees analyzed, social networks played a minor role, as well as economic or social welfare conditions. Basic statistical analyses provided information on specific variables associated with the different routes. Nonetheless, more advanced analyses, including testing of several factors ranging from the characteristics of migrants and motivations for the selection of Germany as a destination to journey-related experiences, could shed more light on the determinants of the formation of different corridors.

Our study underlines the fact that forced migration is a complex and dynamic process with moving targets from the perspective of the people concerned. There is no general pattern underlying the choice of a specific corridor of refuge; neither socio-demographic nor time-related variables explain why certain individuals take a specific route. Many of the Syrians under consideration, who arrived in Germany between 2014 and 2016, traveled via Turkey. Although cultural and linguistic proximity was greater there than in Germany, a reason for continuing onward might have been the prolonged condition for forced migrants of having the status of “tolerated guests” in Turkey, without accountable legal status and living under precarious conditions. As the most recent “Syrian Barometer” in Turkey indicated, the share of Syrian forced migrants in Turkey who were considering moving onward to other countries, mainly in the EU, increased in 2022 (Erdogan, 2022). Millions of Syrian citizens have already lived in Turkey for many years without sustainable conditions concerning their future status. They live in the limbo of uncertainty and lack civic rights as a specifically vulnerable group. Forced migrants in countries like Colombia and Mexico, Jordan and Pakistan, or Uganda and Sudan face similar challenges. Further research should investigate the longitudinal dimension of forced migration more deeply.

This paper adds to the debate on onward migration. Especially in terms of the dynamics of forced migration, it is crucial to understand and explain the conditions under which people tend to return to their country of origin or to move on to other countries. Based on a review of the related literature, we derived specific hypotheses concerning the factors influencing onward migration and the country or countries that forced migrants may choose. The analysis of our survey data suggested that we still do not have a consistent model for understanding and explaining agency and decision-making in the context of forced migration. Given the global situation in regard to increasing volumes and increasingly differentiated types of forced migration, much scientific work still remains to be done.

## Data availability statement

The panel survey of the Institute for Employment Research (IAB), the Research Center of the Federal Office for Migration and Refugees (BAMF-FZ), and the German Socio-Economic Panel (SOEP) at the German Institute for Economic Research (DIW Berlin) is the dataset of this article. The dataset is available by applying to the institutions. The dataset is only available to the scientific community, and all users need to sign a data distribution contract. Requests to access these datasets should be directed to soepmail@diw.de.

## Author contributions

LP contributed to the study's conceptualization and developed the main research questions. All authors contributed to allsections in this article. All authors contributed to the article and approved the submitted version.

## Footnotes

^1^The total number of asylum applications was 2.5 million, with 2.38 million first time applications; see https://ec.europa.eu/eurostat/statistics-explained/index.php?title=Asylum_statistics.

^2^See Bourdieu (1986) for the three types of capital mentioned.

^3^The dataset was collected by a consortium of three leading institutions in Germany: the DIW/Berlin [a scientific research institute that has organized the German Socio-Economic Panel Study (SOEP) since 1984], the BAMF/Nürnberg (the Federal Office for Migration and Refugees), and the IAB/Nürnberg (the Institute for Employment Research of the German Federal Employment Agency); for details of the dataset, see Section 3. This paper was prepared in the context of a research project funded by the German Research Foundation, Pr 637/14-1.

^4^We prefer to use the term “forced migrants” in preference to “refugees” or “asylum seekers,” as the persons we write about might have had quite mixed circumstances of escape and might live under different legal statuses.

^5^Concerning the term “organized violence,” see, e.g., Carayannis (2003), Grawert (2008), Basham (2011), Basedau et al. (2016), Manrique Rueda and Tanner (2016).

^6^Içduygu (2015) mentions the 1934 Settlement Law as an inital legal document on the status of forced migrants and immigrants.

^7^Although the Turkish migration agency DGMM produces statistics on Syrian migrants based on its registration system, comprehensive sociodemographic data on these migrants' profiles are only provided by surveys and scientific research.

^8^In the case of Syrian forced migrants in Germany, we use the term “refugee,” as almost all those who arrived in Germany applied for official asylum status.

^9^Ragab et al. (2017) cite a study by Rich (2016) for these proportions. See Rich (2016).

^10^For details of the survey, see: https://www.diw.de/en/diw_01.c.538695.en/projects/iab-bamf-soep_survey_of_refugees_in_germany.html and https://www.diw.de/en/diw_01.c.616027.en/quality_control_in_the_iab-bamf-soep_survey_of_refugees.html.

^11^For the following data analysis, version soep.v34 was used, see: https://www.diw.de/en/diw_01.c.615977.en/soep.v34.html.

^12^In the data, stays in separate countries during flight were recorded only if they were of a duration of 3 months or more. In order to identify the routes, we created a new variable representing the number of such stays before arrival in Germany. The mean number of such stays in our selected sample was 3.05. We then decided to include the first five stages of movement in our analysis. In the second phase, we generated custom tables of migratory movements for the first five stages. Next, we defined the corresponding region or specific country. Based on custom tables, we then identified 68 combinations of places as specific routes. For instance, the route Syria–Lebanon–Turkey–any EU country–Germany was used by only one migrant, but the route Syria–Turkey–EU–Germany was used by more migrants. In the last phase, we grouped these combinations of places into ten specific routes according to the number of stages of movement.

^13^See, e.g., official Frontex registrations since January 2009: https://frontex.europa.eu/assets/Migratory_routes/2020/Monthly/detections_of_IBC_2020_12_08.xlsx.

^14^See, e.g., https://en.m.wikipedia.org/wiki/Timeline_of_the_Syrian_Civil_War.

^15^Multiple responses to this question were possible. Figure 2 shows the percentage distribution of the answers, the distribution of the respondents regarding to the answers are higher due to the multiple response structure in the question. The percentages as follows: Fear of recruitment was mentioned by 46% of interviewees, while 44% mentioned persecution. Discrimination was selected by 42% of the respondents. Concerning economic reasons, personal living conditions and the economic situation in the country were mentioned by 45 and 38% of the respondents, respectively.

^16^Multiple answers to this question were possible. The totals corresponded to 48, 48, and 30% of the cases, respectively.

^17^These answers were provided in response to a multiple-response type question. The most frequently mentioned responses were reported by 48, 31, and 26% of migrants, respectively.

^18^All percentages reported in the main text represent the share of all responses given. Due to the structure of the multiple-response question, the percentages increase when considering the share of respondents who gave a particular response. In particular, 79% of respondents mentioned respect for human rights as the reason for selecting Germany. The second most frequent answer of the German education system was given by 58 percent of interviewees. A welcoming culture and governmental/social welfare were mentioned by 55 and 38% of migrants, respectively.

^19^To calculate the adjusted residuals, the difference between each observed value and the expected value in the case of no statistically significant association between the variables is first standardized and then adjusted by dividing the result by an estimate of the corresponding standard deviation. The resulting standardized residual reflects the number of standard deviations by which the value falls above or below the mean, where 0 represents the mean of the standardized residual and 1 the standard deviation. Values above 1 or below−1 reflect the weight of the differences between observed and expected values in the cell in terms of explaining the overall association between the two variables.

^20^While the residuals indicate the general trend, the odds ratio describes the trend in terms of how many times more or less likely migrant in a certain group are to use one corridor as opposed to other corridors (see Agresti, 2002, p. 81 and 82).
